# Preference of Diamondback Moth Larvae for Novel and Original Host Plant after Host Range Expansion

**DOI:** 10.3390/insects5040793

**Published:** 2014-10-27

**Authors:** Kathrin Henniges-Janssen, David G. Heckel, Astrid T. Groot

**Affiliations:** 1 Department of Entomology, Max Planck Institute for Chemical Ecology, Hans-Knöll-Str. 8, Jena 07745, Germany; E-Mails: heckel@ice.mpg.de (D.G.H.); a.t.groot@uva.nl (A.T.G.); 2 Institute for Biodiversity and Ecosystems Dynamics, University of Amsterdam, Science Park 904, Amsterdam 1098 XH, The Netherlands

**Keywords:** larval preference, feeding behavior, experience, host strain, *Plutella xylostella*

## Abstract

Utilization of a novel plant host by herbivorous insects requires coordination of numerous physiological and behavioral adaptations in both larvae and adults. The recent host range expansion of the crucifer-specialist diamondback moth (DBM), *Plutella xylostella* L. (Lepidoptera: Plutellidae), to the sugar pea crop in Kenya provides an opportunity to study this process in action. Previous studies have shown that larval ability to grow and complete development on sugar pea is genetically based, but that females of the pea-adapted strain do not prefer to oviposit on pea. Here we examine larval preference for the novel host plant. Larvae of the newly evolved pea-adapted host strain were offered the choice of the novel host plant sugar pea and the original host cabbage. These larvae significantly preferred pea, while in contrast, all larvae of a cabbage-adapted DBM strain preferred cabbage. However, pea-adapted larvae, which were reared on cabbage, also preferred cabbage. Thus both genetic differences and previous exposure affect larval host choice, while adult choice for the novel host has not yet evolved.

## 1. Introduction

The present-day patterns of species diversity of flowering plants and herbivorous insects reflect the ongoing process of co-evolution in which insects and their plant hosts are in a constant arms race [1]. Plants evolve novel substances to defend themselves and escape from the pressure of herbivory and the herbivore, in turn, evolves tolerance to these defenses to explore a novel host plant that provides reduced competition [1,2,3]. In recent times, human activities have influenced this coevolutionary process. Introductions of non-native or agricultural plants into novel habitats without their co-evolved counterparts may induce novel plant-insect interactions [4]. Agro-ecosystems are ecologically simple with a few associated species [5], and the trend towards an increased and accelerated rate of pest species changing their hosts in agro-ecosystems clearly demonstrates the need for understanding such colonization events in non-natural environments [6,7].

In herbivorous insects, the successful use of a host plant depends on specific behavioral and physiological adaptations in the adult and larval stage. In other words, existence of an insect-host plant association requires that adult females find and accept a plant for egg laying and larvae accept it and are able to fully develop on it [8]. Accordingly, the acquisition of a novel plant as host, *i.e.*, the establishment of a novel insect-plant interaction, is presumably governed by changes in behavior by adults or larvae or both. Feeding and/or oviposition behavior have to change from “not being attracted to” or “being repelled from” to “acceptance of” or even “preference for” the novel host. This was the case in the checkerspot butterfly *Euphydryas editha*, which incorporated the introduced plant *Plantago lanceolata* into its diet in the last 100 years [9]. Oviposition preference for the novel host, and rejection of the native host, has since evolved in populations that reside in regions where the introduced plant grows [9,10]. Similarly, hatchling preference in the soapberry bug, *Jadera haematoloma*, has also changed. Ancestral populations of *J. haematoloma* have a preference for feeding and reproducing on the original host plant balloon vine (*Cardiospermum halicababum*), while populations that shifted to the introduced ornamental goldenrain tree (*Koelreuteria* sp.) prefer feeding on the novel host [11].

Behavior plays a central role in the acceptance of a novel host plant and changes in this behavior might be the driver for establishment of novel insect-plant relationships [8,12]. A thorough understanding on the exact changes in feeding and oviposition behavior after a change in host plant is of evolutionary as well as practical relevance. Evolutionarily, the acquisition of a novel host plant can be an important step towards host race formation that might ultimately lead to the formation of a novel species [13]. If attacks of novel host plants by herbivorous insects lead to subpopulations, one specialized on the novel and the other on the original host plant, these may evolve into host races [13,14]. Two major factors are needed for host race formation: host preference and host-associated fitness. Host preference initiates the process of host race formation, and close association with the novel host plant will reduce gene flow between specialized subpopulations [15]. Gene flow will be reduced even more if this host preference is accompanied by fitness consequences in larval performance on the novel host plant [16]. A preference for the novel host plant in adult and larval stages of the herbivore will most likely lead to host race formation, whereas an incomplete change in these behaviors, e.g., one stage still preferring the original host plant or a continued acceptance of original and novel host plant, increases the chance of a backshift to the original. From an applied perspective, a thorough understanding of behavioral changes in larval and adult stages of pest insects when changing host plants is important for implementation of adequate control measures. This knowledge can improve the ability to predict unwanted host range expansions of pest insects.

Recently, the highly specialized crucifer-feeding diamondback moth (DBM), *Plutella xylostella* L. (Lepidoptera: Plutellidae), has been found on a sugar pea field, *Pisum sativum* L. *var*. *macrocarpon*, cultivar Oregon Sugar Pod (Fabaceae), in Kenya in the area south of Lake Naivasha, a region well-known for its intensive export vegetable production [17]. DBM is a significant worldwide pest of cultivated crucifers, and also utilizes wild crucifers as hosts. Crucifers are in the Brassicaceae, a plant family characterized by the glucosinolate-myrosinase defense system against herbivore attack [18]. DBM has the ability to deactivate this defense system using a highly active glucosinolate-sulfatase, and thus is specifically adapted to brassicaceous plants [19]. Therefore it was surprising to find DBM feeding on sugar peas. During that season in 1999, DBM densities on the original cabbage hosts were extremely high, and thus a neighboring pea field became infested. In the following years this local population even expanded to an adjacent field of mangetout peas (*Pisum sativum* L. var. *macrocarpon*, cultivar Snow Green). Because the population persisted as an uncontrollable pest on the pea crop in the following two years, the farmer stopped growing peas, so that this population either became extinct in the field or rejoined the populations feeding on the neighboring cabbage. Larvae were collected from the pea crop in 2000 and 2002, and have been reared on Oregon Sugar Pod peas in the laboratory since then [20]. This DBM population represents a unique example for a very recent expansion from the original plant family (Brassicaceae) to a new and dissimilar host plant family (Fabaceae) in the field and the opportunity to investigate behavioral changes underlying a contemporary event of adaptation to a chemically and evolutionarily unrelated novel host.

In the laboratory, DBM larvae from the pea-adapted population (referred to as DBM-P) can complete their development on pea plants, while DBM larvae of other strains cannot survive on pea plants [20]. Previously, we found that DBM-P adult females continue to prefer ovipositing the original host plant [21]: DBM-P females laid most eggs on cabbage and very few on peas, while they laid significantly more eggs on cabbage plants when pea plants were present. Larval fitness of DBM-P, measured as survival rate, was the same on the novel as on the original host [20]. However, developmental time was increased and pupal weight reduced compared to DBM-P larvae reared on kale [20,21]. Recently, we also showed that the ability of DBM-P larvae to complete development on pea has a polygenic genetic basis [22]. Together, these results suggest that host range expansion in DBM-P is still in the early stages, and raises the question how larval preference is involved in the host choice behavior of this strain, which was the purpose of this study. The characterization of DBM-P’s larval preference, *i.e.*, either for the original or novel host plant, will complete assessment of the behavioral phenotype of this novel strain and the potential of host race formation in DBM-P and help to understand the mechanisms of rapid plant acquisitions in agro-ecosystems.

## 2. Experimental Section

A larval feeding preference assay was set up with fourth instar larvae (L4) of the DBM-P strain. Their feeding preference was recorded by offering pea and cabbage leaf discs and recording the position of the larvae and amount of consumed material per disc after certain time increments in comparison to the choice of larvae from an original cabbage-adapted host strain (DBM-Cj). Using mature L4 larvae introduces the complication that these larvae are already experienced and conditioned to feed on their rearing host plant, *i.e*., DBM-P on pea and DBM-Cj on cabbage. Therefore, a group of DBM-P larvae were additionally reared on cabbage and then tested for their feeding preference on the two host plants, sugar pea and cabbage.

### 2.1. Insects

Two strains of *P. xylostella* were used in the larval feeding choice assay: the cabbage feeding strain (DBM-Cj) and the pea adapted strain (DBM-P). Both strains originate from Kenya and were kindly provided by Bernhard Löhr from the International Centre of Insect Physiology and Ecology (ICIPE), Nairobi, Kenya. DBM-P was originally collected from the infested pea field in Naivasha in 2000, and was repeatedly replenished with additional field-collected material from the same site for the next two years [20]. It has been maintained as a laboratory culture since then at ICIPE in Kenya. DBM-Cj is derived from a field population from the semi-arid areas about 40 km south east of Nairobi. Both strains were sent to the Max Planck Institute for Chemical Ecology (Jena, Germany) in May 2005, where they have been reared for more than 50 generations since then. Population sizes of the strains maintained in Kenya are unknown to us, but averaged about 400 adults per generation in Jena. Insect cultures of both strains were reared from egg to adult stage on intact plants in mesh cages (60 × 60 × 60 cm) at 21 °C, 50% RH, and 16:8 L:D photoperiod, with DBM-Cj reared on cabbage and DBM-P reared on pea. For mating and oviposition, adult moths were collected with an aspirator (BioQuip Products, Rancho Dominguez, CA, USA) and transferred from cages to mating boxes (15 × 15 × 5 cm). Each box contained at least 30 individuals, and per generation 12 to 15 boxes were set up. The bottom of the boxes was covered with tissue paper on which leaves of the respective host plants (cabbage or pea) were placed as oviposition sites. Adult moths were fed with 5% honey solution. After eggs were deposited on leaves and the tissue paper, these were transferred to cages and fresh leaves were added to the plastic box. For this study, we additionally reared DBM-P larvae on cabbage, referred to as DBM-Pc, under same conditions as DBM-P and DBM-Cj.

### 2.2. Plants

Seeds of pea, *Pisum sativum* L. var. *macrocarpon*, cultivar Oregon Sugar Pod, were obtained from Agri-Saaten GmbH (Bad Essen, Germany). Cabbage seeds, *Brassica oleracea* var. capitata, cultivar Gloria, were obtained from B and T World Seeds (Aigue-Vives, France). Plants used for rearing of insects were grown in trays (58 × 32 × 11.5 cm) in Klassmann Tonsubstrat under greenhouse conditions at 21–23 °C, 50%–60% RH and 14:10 L:D photoperiod. For larval choice experiments, five-week-old pea and cabbage plants were used.

### 2.3. Larval Feeding Choice Assay on Leaf Discs

**Figure 1:**
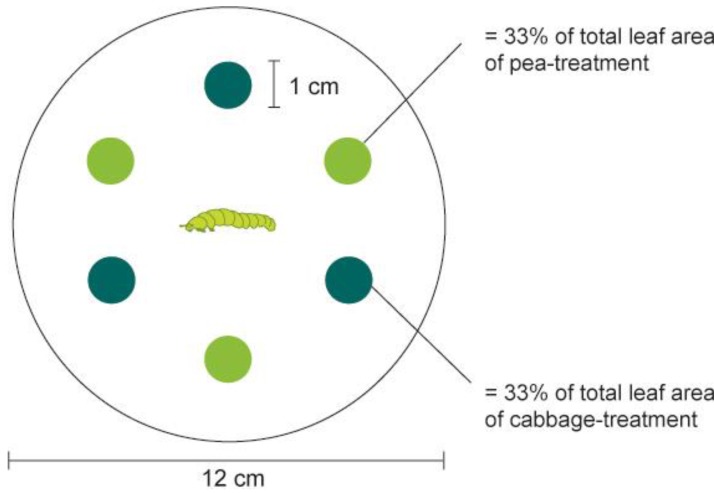
Experimental set-up of larval feeding choice assay. Pea and cabbage leaf discs were offered in an alternate order and a fourth-instar larva was placed in the middle of the Petri dish. The positions of larva were recorded after 5 min, 30 min, 6 h and 12 h, and percentage of consumed leaf area was recorded after 30 min, 6 h and 12 h, respectively.

The set-up of the larval feeding choice experiment is depicted in Figure 1. We used pea and cabbage leaf discs (1 cm diam) that were punched from the leaves of plants grown under the above-described conditions. The discs were placed in a Petri dish (12 cm diam). Larvae crawling on the bottom of the Petri dish could easily reach a disc and begin feeding. A filter paper moisturized with 1 mL of H_2_O was placed on the bottom of the Petri dish to prevent desiccation of the plant material. Three leaf discs from the two host plants tested, pea and cabbage, were arranged in alternating order (*i.e.*, in total six leaf discs for each arena). This kind of arrangement increased the probability that a larva leaving a disc would encounter the other plant before coming again to the first plant.

Prior to the experiment, larvae were starved for two hours. At the beginning of the test, one fourth-instar larva (L4) of *P. xylostella* was placed in the middle of each arena. An assay consisted of two recordings, the behavioral choice of the larva (*i.e*., location of the larva) and visual estimates of the amount of surface eaten (the percentage of one disc consumed at four time points (after 5 min, 30 min, 6 h and 12 h). The bioassay was replicated 40 times (total of tested larvae = 40) with each DBM strain, DBM-P, DBM-Cj and DBM-Pc. Replicates in which larva left the arena and escaped from the Petri dish were not recorded and excluded from data analysis.

### 2.4. Data Analysis

Data was analyzed using R [23]. The number of larvae choosing either of the two host plants, cabbage and pea, respectively, was counted and compared with a Fisher’s exact test. The percentage of consumed leaf area per strain and time point were compared using a non-parametric Wilcoxon signed rank test. One-way analysis of variance (ANOVA) was used to test for significant differences in the percentages of consumed leaf area between strains after 6 h and 12 h, respectively. The Tukey’s honestly significant difference (HSD) test was used for multiple comparisons.

## 3. Results

**Figure 2:**
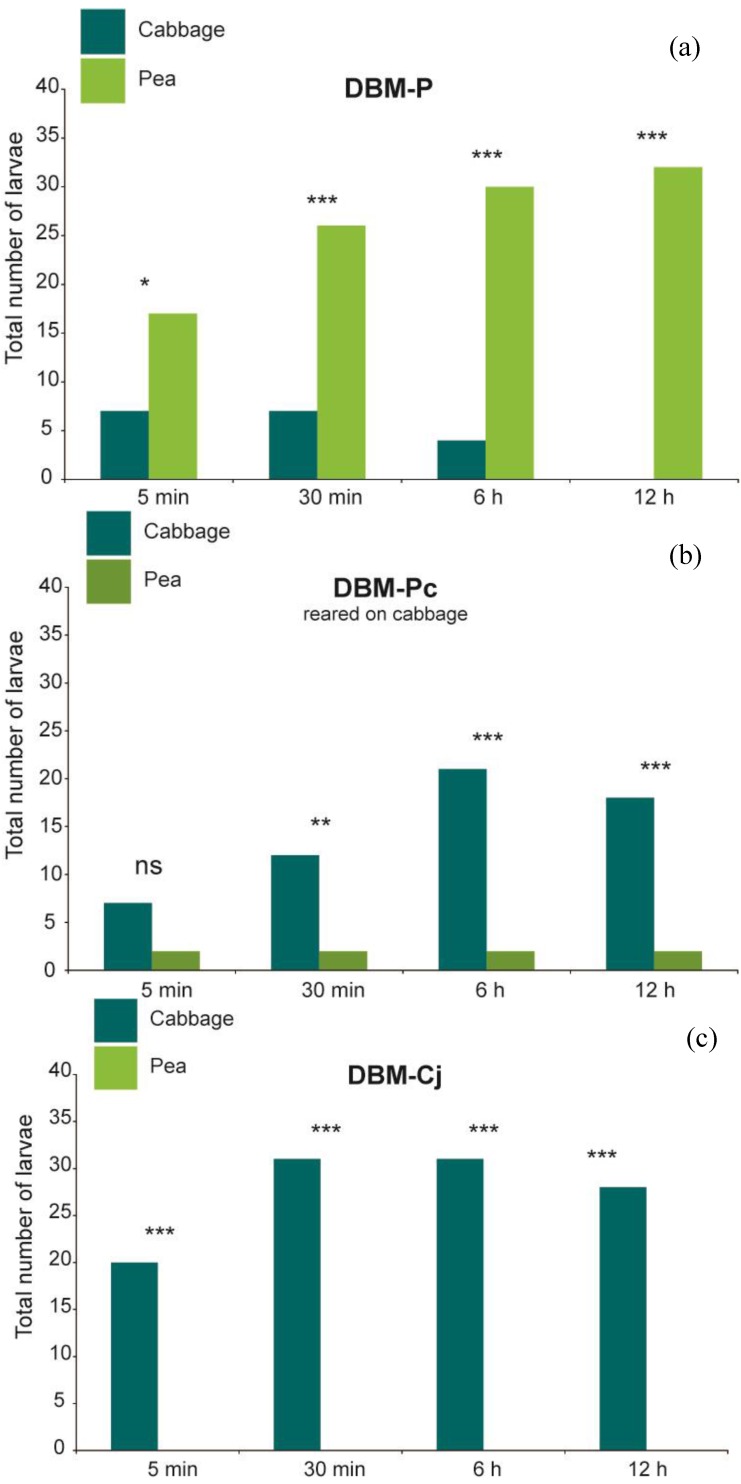
(**a**) Orientation choice of L4 larvae of DBM-P (*n* = 38); (**b**) DBM-P reared on cabbage (*n* = 30); (**c**) DBM-Cj (*n* = 40) after 5 min, 30 min, 6 h and 12 h. ** *p* < 0.01, *** *p* < 0.001, ns = not significant.

**Figure 3:**
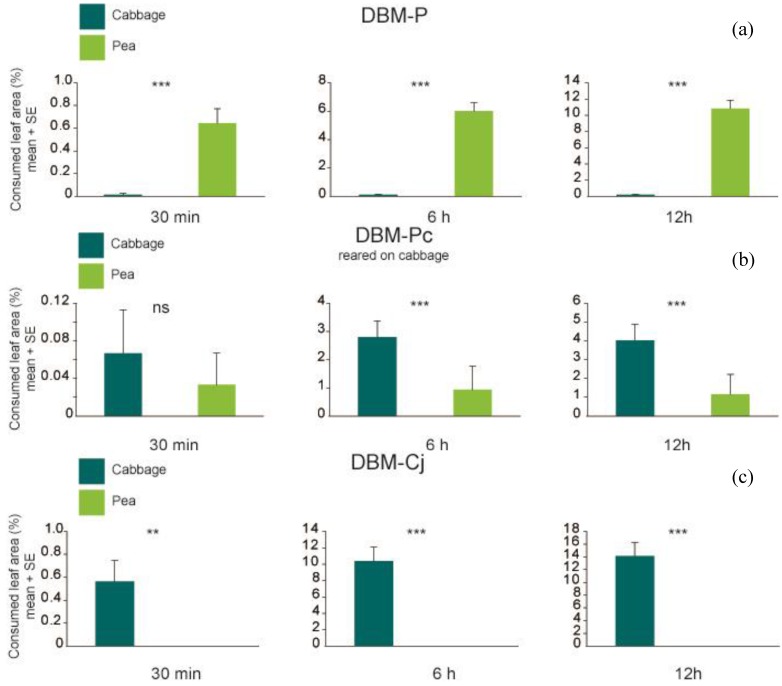
Percentage of consumed leaf area per larva of (**a**) DBM-P (*n* = 38); (**b**) DBM-P reared on cabbage (*n* = 30) and (**c**) DBM-Cj (*n* = 40) after 30 min, 6 h and 12 h. Symbols and abbreviations as in Figure 2.

At all observation times, larvae of DBM-P were recorded significantly more often on their novel host plant pea than on cabbage (Figure 2; Fisher’s exact test *p* = 0.024 at 5 min and *p* < 0.001 at 30 min, 6 h and 12 h), and consumed significantly more pea than cabbage leaf discs (Figure 3; Wilcoxon signed rank test *p* < 0.001 at all observation times). In contrast, DBM-Cj larvae were found significantly more frequently on cabbage leaf discs (Figure 2; Fisher’s exact test and *p* < 0.001 at all observation times) and exclusively consumed cabbage, so that the percentage of consumed cabbage was significantly higher than that of pea (Figure 3; Wilcoxon signed rank test *p* = 0.005635 at 30 min and *p* < 0.001 at 6 h and 12 h). DBM-Pc spent significantly more time on cabbage only at the last three time points (Figure 2; Fisher’s exact test *p* = 0.0017 at 30 min and *p* < 0.001 at 6 h and 12 h). The amount of cabbage or pea leaf discs eaten was not significantly different at the first time points (Wilcoxon signed rank test *p* = 0.7728), but significant at the later time points of 6 h and 12 h (Wilcoxon signed rank test *p* < 0.001 at 6 h and 12 h). Total amount of leaf area consumed by DBM-P larvae differed significantly from the amount consumed by DBM-Cj larvae (P_adj_ = 0.01), but not from DBM-Pc (P_adj_ = 0.37) after 6 h. After 12 h, total amount of leaf area consumed by DBM-P larvae did not differ from the amount consumed by DBM-Cj larvae (P_adj_ = 0.30) or by DBM-Pc (P_adj_ = 0.06).

## 4. Discussion

The case of DBM feeding on sugar pea in Kenya is an example of the recent trend of increased change in interactions between insects and their plant hosts due to human disturbances, e.g., intensified agriculture. Although agro-ecosystems have low ecological diversity, they are closely monitored and thus provide the opportunity to detect events such as the invasion of a novel pest insect by host shift or range expansion. Thus, the availability of background information on the circumstances of host shifts in agro-ecosystems bears an advantage over studies that evaluate host plant changes from the evolutionary past, which often can only infer range expansions and shifts underlying present host plant relationships. This was also true for DBM’s host range expansion, for which was known that it occurred in a situation where the original host plant was extremely rare due to severe DBM attack, so that a neighboring sugar pea field was the only available and reachable green food source for DBM. The shift to the neighboring pea field and adaptation to the novel host occurred rather rapidly and therefore specific features of the newly evolved strain could be anticipated, e.g., a stage of on-going host plant adaptation.

In this study we demonstrated that DBM-P larvae preferred their novel host plant sugar pea. Thus, preference in the larval stage differs from that in adult stage, as females of DBM-P did not show any oviposition preference for pea except for an increased oviposition rate on cabbage in the presence of pea [21]. DBM-P has expanded its host range to sugar pea in the field and is raised on its novel host in the laboratory. Despite this adaptation, we have previously shown that DBM-P still develops slower on pea than a cabbage strain develops on cabbage, and pupal weight of DBM-P also is lower on pea [20,21]. In the current tests, DBM-P consumed less leaf area within 6 h than did DBM-Cj, but the amount of total consumed leaf area was not different after 12 h. Thus, the choice that allows DBM-P larvae to utilize a resource that DBM-Cj cannot, seems to result in a moderately reduced performance relative to the original host plant. Whether this reduced performance is due to an insufficient physiological adaptation to be able to cope with defense and secondary plant compounds of sugar pea is not known.

Although it was not surprising to find all but two DBM-Cj larvae exclusively feeding on cabbage, a few more cases of at least larval feeding initiation on pea would have been expected. It has been shown that a cabbage-adapted strain can be selected for survival on pea within six generations, a selection that results in extremely high mortality in the early generations [20]. Thus, some genetic variation for larval preference may exist, even in typical cabbage-adapted strains. Genetic analysis of the ability to complete development on pea has further shown that DBM-P still possesses genetic variation for this ability [22]. Therefore, the host range expansion likely evolved from existing standing genetic variation instead of from a spontaneous mutation, which allowed for such a rapid switch to an unrelated host plant. The prior conditioning of DBM-Cj larvae on cabbage may have masked a preference of some larvae for pea in the choice experiments.

Since the behavior of fourth instar larvae might be affected by prior experience, DBM-P larvae raised on cabbage (DBM-Pc) were also tested. Because all DBM-Cj neonates died when provided only pea, the reciprocal test could not be performed. Preference behavior of DBM-Pc larvae was similar to that of DBM-Cj: larvae were recorded significantly more often on cabbage and also consumed a significantly higher amount of cabbage than pea, showing that prior experience indeed affects choice behavior. Preference induction through learning is a well-known phenomenon studied in a range of phytophagous insects and usually produces a long-lasting behavioral effect [8,24]. For DBM-P larvae, choosing cabbage as host is beneficial because their development on cabbage is faster than on pea [20]. However, preference of DBM-Pc for cabbage was not as strong as that of DBM-Cj: in contrast to DBM-Cj, a few DBM-Pc larvae chose pea and fed on it, suggesting a conflict between innate preference and experience. Also, DBM-Pc larvae significantly preferred cabbage after 30 min, while DBM-P and DBM-Cj larvae showed a significant preference for pea and cabbage, respectively, already at 5 min; and DBM-Pc consumed smaller amounts of cabbage than did DBM-Cj of cabbage and DBM-P of pea. Together, these results indicate that despite pre-conditioning on cabbage, preference for pea was maintained in some DBM-Pc larvae, and those larvae with a preference for cabbage took longer in their decision-making and showed restricted consumption.

To further investigate the interaction of pre-conditioning with innate preference, newly hatched first instar larvae with no prior host plant experience should be tested. A possible complication in interpreting those experiments is the possibility that neonate larvae may have a tendency to accept the first encountered host plant on which they initiate feeding and which they rarely leave because of their restricted mobility [25]. Therefore, second and third instar larvae should also be tested to see whether a conditioning effect increases with the total exposure time to a given host.

The available information on larval fitness and female preference together with the herein obtained results on larval preference provides the necessary data to address the question whether the behavioral changes that governed the host range expansion are sufficient for host race formation in DBM. Host race formation has been studied in a variety of insect species, and so far the most thoroughly studied example of host race formation is that of the apple maggot fly *Rhagoletis pomonella* [26], which quite recently switched to apple (*Malus* sp.) from the native hawthorn (*Crataegus* sp.). Among other traits, host races on apple and hawthorn differ in host preference [27] and phenology [28] on the two hosts but not in survival rates under laboratory conditions. DBM-P has just initiated the first steps in a range expansion towards sugar pea, and because DBM-P still prefers the original host plant for oviposition and larvae perform better on the original host a backshift is very likely. Adult females are the mobile life stage and predominantly determine the host plant that the larvae must use, or die [29]. Moreover, although larvae may prefer the novel host they are unlikely to leave a cabbage plant and move to a patch with sugar pea plants due to their limited dispersal ability [25], except in a case of severe deprivations of the original cabbage host plant. Host race formation in theory could result from a host range expansion, but DBM-P currently appears to lack the complete set of necessary features for this transition.

DBM’s recent host range expansion to pea also has implications for its pest status. This insect is already known to be able to thrive in almost every climate as well as to develop resistance against every insecticide [30,31]. With these characteristics it is considered one of the most severe pest insects worldwide [18]. Sudden host range expansions to economic important crop species unrelated to the original host crucifers, such as to sugar pea in Kenya, have so far not been reported. The ability to rapidly adapt to unrelated plant species is thus a novel feature in this pest insect. An intensified agriculture and increased numbers of introductions of crop species has likely increased the opportunities for such host switches in recent years. It seems that in DBM larval preference changes more rapidly than adult preference. However, although DBM-P larvae preferred the novel host plant, they have not yet lost the ability to survive equally well on the original host and even prefer it when raised on it. Thus, although opportunities for range expansions may increase, it is not expected that range expansion is followed by host race formation. Host range expansions are likely to be only short-term, for maintenance of the population and the availability of the (preferred) original host will probably lead to a backshift. Employing a similar strategy, DBM temporarily establishes on wild crucifers as alternative refugia in order to escape insecticidal treatments or to maintain populations outside the growing season [18,32]. A thorough understanding of the underlying behavioral changes in DBM-P might have prevented the farmer from completely stopping growing sugar pea, but instead implementing control measures to eradicate the larvae with a preference for pea. With females not ovipositing on sugar pea, the pest would then have shifted back to the original host plant itself.

## 5. Conclusions

While DBM-P females still prefer to oviposit on cabbage and presence of sugar pea only increases the number of eggs laid on cabbage, DBM-P larvae significantly prefer their novel host. When these larvae are reared on cabbage, they prefer their rearing host cabbage, although some larvae showed a preference for pea and still fed on it. These behavioral changes most likely enable DBM to establish on the novel host plant pea, but still represent only a partial transition to formation of a novel insect-plant interaction.
